# Time-sensitive effects of quercetin on rat basophilic leukemia (RBL-2H3) cell responsiveness and intracellular signaling

**DOI:** 10.1371/journal.pone.0319103

**Published:** 2025-02-24

**Authors:** Mana Matsuo, Shuang Liu, Haruna Yamada, Erika Takemasa, Yasuyuki Suzuki, Masaki Mogi

**Affiliations:** 1 Department of Pharmacology, Ehime University Graduate School of Medicine, Shitsugawa, Toon, Ehime, Japan; 2 Department of Anesthesiology, Saiseikai Matsuyama Hospital, Matsuyama, Japan; 3 Research Division, Saiseikai Research Institute of Health Care and Welfare, Tokyo, Japan; Universidade Federal do Rio de Janeiro, BRAZIL

## Abstract

Quercetin is known for its ability to inhibit mast cell degranulation and reduce the release of inflammatory mediators. However, it has also been reported to sensitize mast cells, potentially leading to hyperresponsiveness. This necessitates careful optimization of its use in the treatment of chronic inflammatory diseases. To fully harness quercetin’s therapeutic potential, this study investigated the effects of quercetin on rat basophilic leukemia (RBL-2H3) cells responsiveness over varying durations of exposure. We employed comprehensive transcriptome analysis and subsequent functional validation of key signaling pathways. Our findings revealed that quercetin initially reduced cell activity with short-term treatment. However, with prolonged exposure, quercetin transiently enhanced both IgE cross-linkage-mediated and non-IgE-mediated responses. Specifically, prolonged quercetin treatment downregulated IgE-mediated degranulation and FcεRI expression, while potentially sensitizing RBL-2H3 cells to other non-IgE secretagogues through enhanced PKC activity. Given quercetin’s multifaceted effects on intracellular signaling pathways, it is crucial to further investigate its efficacy and potential risk of adverse effects. Future studies should focus on a deeper understanding of these mechanisms to optimize quercetin’s therapeutic applications while mitigating any possible negative outcomes.

## Introduction

Quercetin (3,3’,4’,5,7-pentahydroxyflavone), a ubiquitous flavonoid class of polyphenols contained in various fruits and vegetables, has been widely recognized as an anti-inflammatory agent, and has been shown to reversibly inhibit stimulatory signals and release of inflammatory mediators in mast cells, which are a key source of mediators responsible for acute allergic reactions, and other immune cells involved in adaptive immune responses [1,2]. The capacity of quercetin to down-regulate IgE-dependent intracellular regulatory signaling events initiated by FcεRI cross-linking enables its usage for the treatment of acute and chronic inflammatory conditions such as asthma [2,3]. Previous exploratory studies indicate that the anti-inflammatory properties of quercetin may involve physiological functions through crosstalk among the phospholipase C (PLC)/protein kinase C (PKC) cascade for secretion of granules and the ERK cascade for activation of phospholipase A2 and the release of arachidonic acid, and other signaling networks [4].

In addition to adaptive immunity, mast cells can undergo activation or, in some cases, suppression, by a variety of signals that can be generated during innate immune responses. Activating signaling includes components of complement activation independently of antibodies; agonists of Toll-like receptors; adenosine; corticotropin releasing factor receptors type 1; a variety of endogenous peptides including substance P and VIP; and many other stimuli including physical stimulation [5,6]. According to previous reports, prolonged exposure to quercetin may be capable of modulating the capacity of the responsiveness of mast cells to these secretagogues related to innate stimuli [7]. Transient activation of the G_i_ protein/PLC/PKC signaling cascade could be a potential mechanism underlying the cellular response to quercetin, associated with rapid secretion of granules and release of arachidonic acid.

Given quercetin’s well-documented anti-inflammatory properties and its potential role in modulating mast cell activity in innate immune response), it is crucial to optimize its use in the treatment of chronic inflammatory diseases. To fully harness the therapeutic potential of quercetin, a comprehensive investigation into the responses of mast cells to quercetin treatment over varying durations was conducted. This involved utilizing transcriptome analysis to elucidate changes in the transcriptomic landscape related to intracellular signaling pathways. Subsequent functional validation of key molecular targets identified through this analysis enabled a deeper understanding of quercetin’s efficacy and highlighted the potential risk of adverse effects. These findings provide valuable insights into the optimized use of quercetin for chronic inflammatory disease management.

## Materials and methods

### Cells and treatment

Rat basophilic leukemia-2H3 (RBL-2H3) cells (passage 9~42), a well-established mast cell line, were cultured in Eagle’s minimum essential medium (MEM) with 20% fetal bovine serum (FBS) and 1% penicillin/streptomycin, and the cells were maintained at 37°C in a humidified, CO₂-controlled (5%) incubator. For pretreatment with quercetin, the indicated concentrations of quercetin (0.03 – 30μM) were added to the completed culture medium for the indicated pre-incubation durations (1 hour – 2 weeks). Cell viability and proliferation were monitored using a Cell Counting Kit 8 (WST-8/CCK8) (Dojindo Laboratories, Kumamoto, Japan).

### Measurement of capacity of hexosaminidase and histamine release

For release of hexosaminidase, a marker of functional exocytosis in mast cells, RBL-2H3 cells with or without pretreatment with quercetin were seeded at a density of 8 × 10^4^ cells/well in PIPES buffer (pH7.2), containing 25 mM PIPES, 119 mM NaCl, 5 mM KCl, 5.6 mM glucose, 0.4 mM MgCl_2_, 1 mM CaCl_2_, 40 mM NaOH, and 0.1% bovine serum albumin, on a 96-well plate 24 h before activation with secretagogues [8]. To trigger IgE-mediated activation, RBL-2H3 cells were sensitized using 0.5 μg/ml monoclonal anti-2,4-dinitrophenylated bovine serum albumin (DNA-BSA)-IgE for 24 h and stimulated with 20 ng/ml DNP-BSA for 30 min at 37 °C. Thapsigargin (TG) (0.5 μM) or phorbol myristate acetate (PMA)/ionomycin (Iono) (1 μM) was also applied to the cells as a secretagogue for 30 min at 37 °C.

The collected supernatant was incubated with 2.5 mM ρ-nitrophenyl-2-acetamido-deoxy-β-D-glucopyranoside and 50 mM sodium citrated buffer (pH 4.5) at 37 °C for 1 h. The reaction was stopped by application of 2 M potassium hydroxide. Liberated ρ-nitrophenol was measured using a microplate reader at 405 nm.

For measurement of histamine release, RBL-2H3 cells with or without pretreatment with quercetin were seeded at a density of 5 × 10^5^ cells/well in PIPES buffer 24 h before subsequential stimulation. After the cells were activated by indicated secretagogues, the amount of histamine in the supernatant or lysed cells was measured by a high-performance liquid chromatography system, as previously described [9]. *O*-phthaladehyde-labelled histamine was detected using an EICOM HTEC 500E electrochemical detector (EICOM, Kyoto, Japan).

### RNA-seq and bioinformatic analysis

Total RNA was isolated from RBL-2H3 cells using a NucleoSpin RNA Plus kit (TaKaRa, Tokyo, Japan) after 0, 1, or 24 h of quercetin (3 μM) pre-treatment. After RNA quality assessment on a Bioanalyzer 2100 system (Agilent Technologies, Tokyo, Japan), samples with RIN value of 8.0 or higher were selected for subsequent preparation of a library. Nanopore sequencing was carried out using a ligation-based cDNA-PCR sequencing kit (Oxford Nanopore Technologies, Didcot, UK) according to the manufacturer’s protocol, as previously described [10]. Briefly, using a strand-switching protocol, full-length cDNA from 50 ng total RNA was prepared using Maxima H Minus Reverse Transcriptase (ThermoFisher Scientific). Full-length transcripts were amplified using LongAmp Taq 2x Master Mix (New England Laboratories, Tokyo, Japan). The adaptor-ligated libraries were sequenced using a MinION sequencer with FLO-MIN 106 flow cells and R9.4 chemistry. Basecalling was performed using a Guppy basecaller (version 6.4.6+ ae70e8f) by translating ionic signals into a nucleotide sequence.

Raw bead counts were obtained, and identification of differentially expressed genes (DEGs) was performed using a cloud platform for integrated differential expression and pathway analysis (iDEP1.1; http://bioinformatics.sdstate.edu/, accessed on 8 February, 2024) [11] by extraction with an FDR cutoff of 0.1 and min-fold change of 2 as the default settings. Gene networks were generated and functional analyses were performed using IPA (QIAGEN Inc., https://www.qiagenbioinformatics.com/products/ingenuity-pathway-analysis) with the default parameter settings [12].

### Measurement of Ca^2+^ influx

Upon activation, intracellular Ca^2+^ was labeled with Fura-2 AM (Dojindo) and dynamic Ca^2+^ influx was monitored with a fluorometric imaging plate reader (FlexStation II, Molecular Devices, Tokyo, Japan) using a 340/380 nm excitation and 510 nm emission set [13]. Briefly, total assay time was 700 s, including an initial 100-s reading window to measure baseline fluorescence level before application of any compound. The plates were read for an additional 300 s after application of 0.5 μM TG. Extracellular Ca^2+^ (2 mM) was then applied to the cells and the plates were read for an additional 300 s. The peak of the ratio of relative fluorescence units (RFU) (340 nm/380 nm) and initial rate of Ca^2+^ influx (in the first 15 s after extracellular Ca^2+^ addition) were calculated.

### Western blotting

Quercetin-pretreated or non-treated RBL-2H3 cells were homogenized in RIPA buffer (ThermoFisher Scientific) with HALT protease and phosphatase inhibitors (ThermoFisher Scientific). The protein extract was separated using a Jess system (ProteinSimple, San Jose, CA), using a 12–230 kD separation module and an anti-rabbit detection module, according to the manufacturer’s instructions [Watanabe, 2024, Amelioration of oxygen-induced retinopathy in neonatal mice with fetal growth restriction]. Polyclonal rabbit anti-FcεRI antibody (E6D9L) (Cell Signaling Technology, Danvers, MA) was used as the primary antibody. The areas of target peaks were normalized by total protein within the same capillary using a RePlex feature and total protein assay (ProteinSimple). Data analysis was performed using Compass software for Simple Western ver.4.1.0 (ProteinSimple).

### PKC activity assessment

Intracellular PKC activity in RBL-2H3 cells was measured using a PKC Kinase Activity Assay Kit (Abcam, Tokyo, Japan), according to manufacturer’s instructions [Ren, 2023, Temperature-induced embryonic diapause in chickens is mediated by PKC-NF-κB-IRF1 signaling]. Briefly, cell lysates were incubated with a synthetic PKC specific substrate for 90 min at 30 °C after addition of ATP. The phosphorylated substrate was recognized by a polyclonal antibody. An HRP-conjugated secondary antibody was then applied, and the assay was finally developed using a TMB substrate and measured in a microplate reader at 450 nm.

### Statistical analysis

All statistical analyses were performed using GraphPad Prism 10 (GraphPad Software, La, Jolla, CA). Comparisons between two groups were performed using unpaired non-parametric Mann-Whitney test. Non-linear regression fit methods were used for dose-dependent response analysis. Data were presented as mean ± standard error. Differences with *P* <  0.05 were considered statistically significant.

## Results

### Prolonged treatment with quercetin restored activation of RBL-2H3 cells

**Fig 1:**
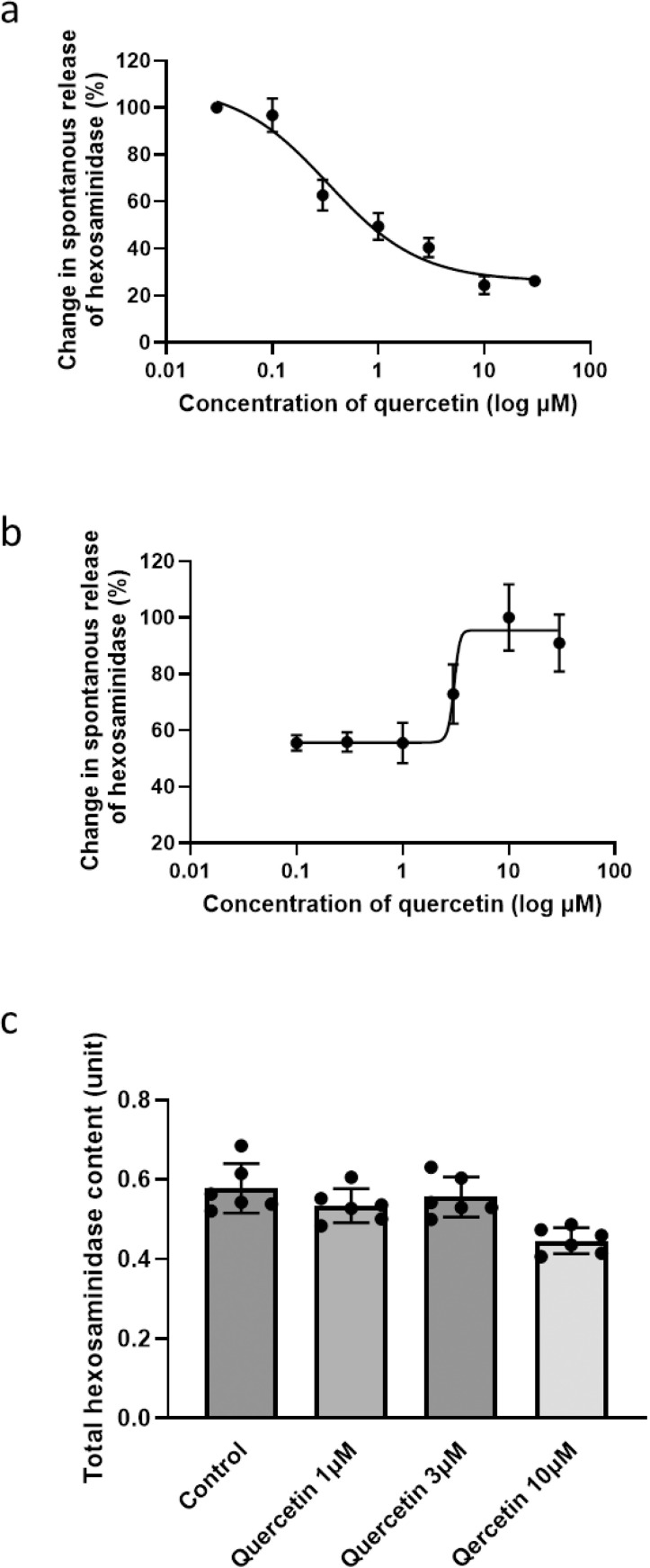
Prolonged treatment with quercetin stimulated activation of RBL-2H3 cells. (a) Quercetin titration curve in thapsigargin-triggered RBL-2H3 cells for short-term (I hour) treatment duration. (b) Quercetin titration curve in thapsigargin-triggered RBL-2H3 cells for prolonged treatment duration (24 hours). The level of released hexosaminidase from non-treated cells was used for normalization, and nonlinear regression fit was performed. Changes in spontaneous release of hexosaminidase are presented as mean ±  S.D. (n =  7). (c) Hexosaminidase content in quercetin-pretreated RBL-2H3 cells for 24 hours at indicated doses. Data are presented as mean ±  S.D. (n =  6).

The initial screening for the influence of quercetin treatment on activation of RBL-2H3 cells with TG as a secretagogue was monitored using a granule marker, hexosaminidase. As expected, one-hour pretreatment with quercetin successfully suppressed the capability of exocytosis in RBL-2H3 cells in a dose-dependent manner, with *IC*_*50*_ of 0.34 μM (Figs 1a and S1). Interestingly, after 24-h pretreatment, a low concentration of quercetin (0.1–1 μM) stably maintained the inhibitory effects to 55.65–59.36% of maximal response, while treatment with relatively high concentrations of quercetin (3–30 μM) restored TG-induced exocytosis dose-dependently (Fig 1b). No significant influence on the total amount of intracellular hexosaminidase was observed in 0, 1, 3, and 10 μM quercetin-treated RBL-2H3 cells (Fig 1c), which indicated that the change in release of hexosaminidase was dependent on the degranulation process. Distinct to short-term treatment, prolonged treatment with quercetin (3–30 μM) for 24 h restored activation of RBL-2H3 cells, with *EC*_*50*_ of 3.03 μM. The dosage of 3μM was therefore used for subsequential prolonged quercetin exposure experiments.

### Transcriptome changes were induced in a time-dependent manner by quercetin treatment in RBL-2H3 cells

**Fig 2:**
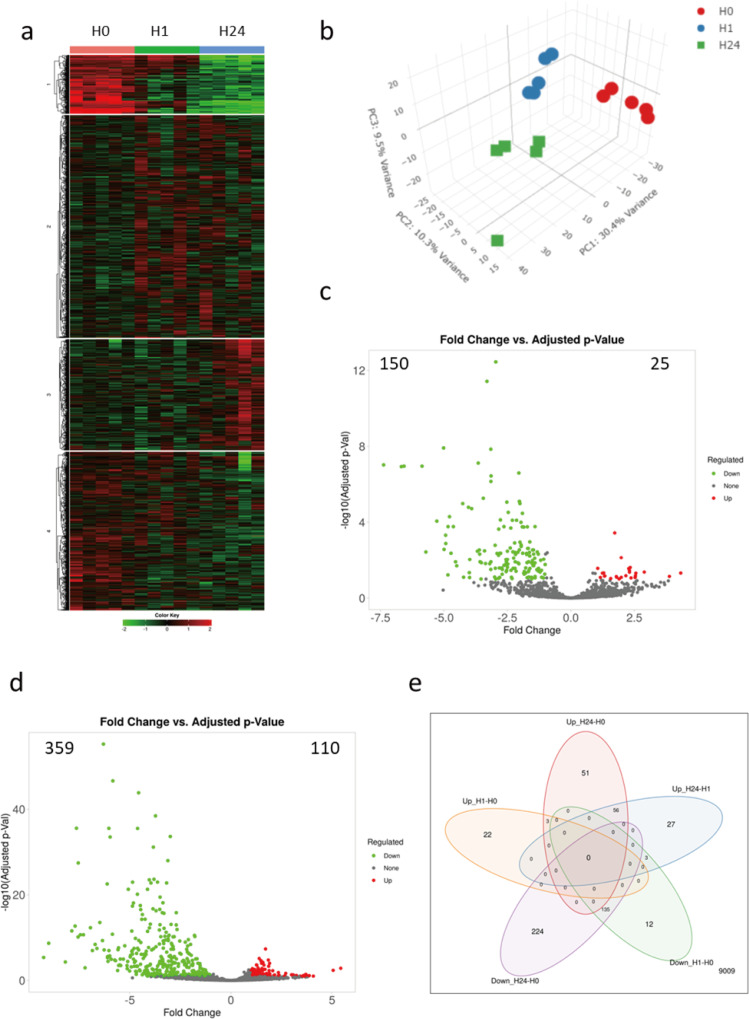
Transcription analysis in 1-hour and 24-hour quercetin-treated RBL-2H3 cells. (a) Heatmap and and k-means clustering revealed four major clusters of the top 2000 expression of genes in non-treated (H0), 1-hour quercetin-treated (H1), and 24-hour quercetin-treated (H24) cells. Expression is displayed as log_2_ (fold-change). (b) Plot of three-dimensional principal component analysis based on first three principal components. The expression of genes is clustered into the experimental group with indistinct data signatures. Volcano plot of differentially expressed genes between non-treated and quercetin-treated cells under (c) short-term (1 hour) and (d) prolonged (24 hour) treatment duration. Significantly downregulated genes are green, upregulated genes are red, and non-significant genes are black. (e) Venn diagram showing numbers of upregulated and downregulated genes in comparisons of 1-hour quercetin-treated vs. non-treated cells and 24-hour quercetin-treated vs. non-treated cells identified by RNA-seq.

RNA-seq technology was utilized to obtain the initial impact on the changes in the transcriptomic landscape of mast cells following varying durations of treatment. Heatmap analysis of top 2000 up-regulated and down-regulated gene in 1h-quercetin-treated, 24h-quercetin-treated, and non-treated RBL-2H3 cells indicated the alteration of the transcriptome landscape in mast cells (Fig 2a). These genes functionally belonged to four clusters, and the results of principal component analysis (PCA) showed that samples were clustered by the duration of treatment based on their transcriptomic profiles (Fig 2b). Among the differentially expressed genes (DEGs), 25 up-regulated and 150 down-regulated genes were extracted from 1h-quercetin-treated versus non-treated transcriptomic comparison, whereas 110 up-regulated and 359 down-regulated genes were extracted from 24h-quercetin-treated versus non-treated transcriptomic comparison (Fig 2c and 2d). One-hour quercetin-treated mast cells and 24h-treated cells shared 3 up-regulated genes and 135 down-regulated genes (Fig 2e).

**Fig 3:**
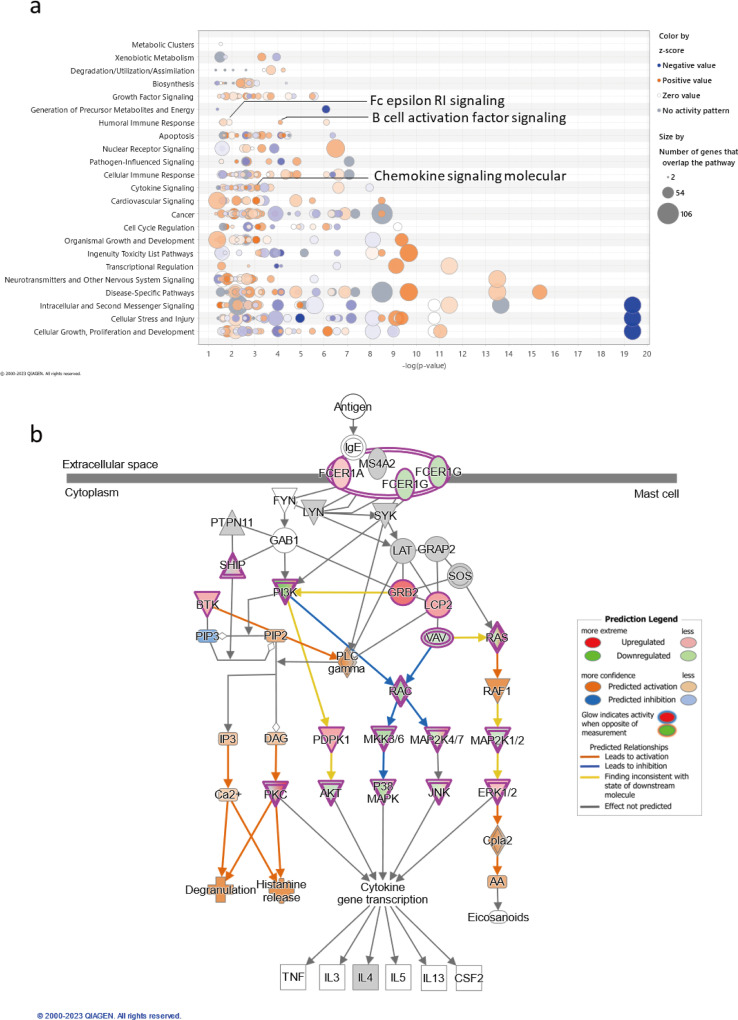
Potential influences of 24 hour treatment with quercetin of RBL-2H2 cells on systemic biological processes. Differentially expressed genes were extracted from the dataset, and pathway analysis was conducted using ingenuity pathway analysis (IPA). (a) Bubble plot of top categories of canonical pathways as z-score (cutoff ≥1.3), which represents a measure of the predicted direction of the pathway activity (24-hour-treated vs. non-treated cells). (b) Fc epsilon RI signaling pathway was identified by IPA. The partial canonical pathway map is overlaid by measurements of fold change in DEGs obtained from RNA-seq analysis and the predicted upstream and downstream effects on other molecules.

To reveal the potential functional influence of prolonged quercetin exposure on mast cells, pathway analysis was conducted and potential regulated biological processes were extracted using the DEGs of 24h-treated and non-treated comparison (Fig 3). Comprehensive functional pathways were affected by prolonged quercetin treatment, including metabolic-related pathways, innate and adaptive immune pathways, growth and development, and some tissue or disease-specific pathways (Fig 3a). Notably, specific pathways that are crucial for sensitization and responsiveness of mast cells were significantly up-regulated in 24h quercetin-treated cells compared to non-treated groups, including FcεRI signaling, B cell activation factor signaling and chemokine signaling molecular pathways. Focusing primarily on the FcεRI signaling cascade, but not limited to it, the gene expression profile indicated that upregulation of the intracellular Ca^2+^ signaling pathway and altered regulation of PKC activity may contribute to the increased degranulation and elevated levels of immunological mediators observed in mast cells subjected to prolonged quercetin treatment (Fig 3b). Since the predicted cellular functions aligned with observations from TG-induced activation, we subsequently verified these potential underlying mechanisms in further studies.

### Distinct effects of quercetin on intracellular Ca^2+^ signaling with varied treatment duration

**Fig 4:**
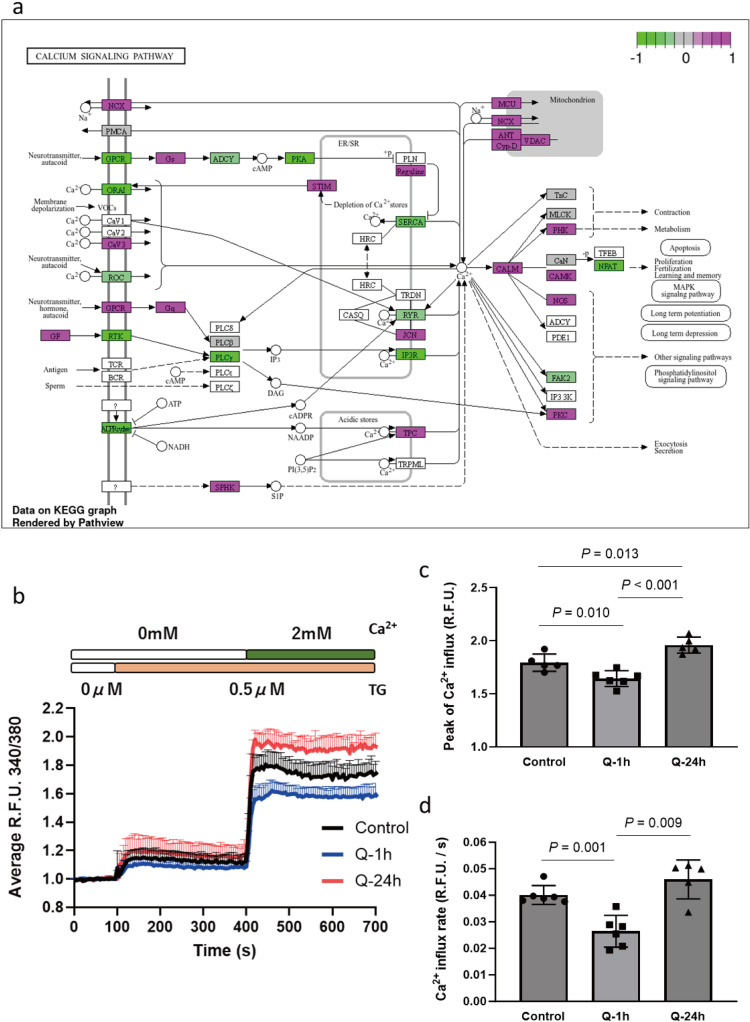
Distinct effects of quercetin on Ca^2+^ influx in RBL-2H3 cells in a treatment duration-dependent manner. (a) KEGG pathway analysis was performed, and the gene expression pattern of the calcium signaling pathway is shown (24-hour quercetin treated vs. non-treated cells). Magenta: upregulated genes; green: downregulated genes. (b) Typical Ca^2+^ influx patterns of non-treated, 1-hour quercetin-treated and 24-hour quercetin-treated RBL-2H3 cells. (c) Peak and (d) rate of Ca^2+^ influx in initial 15 seconds were quantified. Results are expressed as mean ± S.D. (n = 5).

To obtain the initial impact of quercetin treatment on the intracellular Ca^2+^ modulation system in mast cells, we first examined the gene expression profile of the KEGG calcium signaling pathway. In non-excitable cells, store-operated Ca^2+^entry, modulated by the SERCA/STIM/ORAI pathway, plays a critical role. We colorimetrically mapped the changes in gene expression in RBL-2H3 cells treated with quercetin for 24 hours onto the calcium signaling pathway (KEGG) to elucidate the molecular mechanisms involved (Fig 4a). Since it is challenging to determine the effect of quercetin on intracellular Ca^2+^ modulation based solely on the gene expression map, we subsequently conducted a functional assay to evaluate Ca^2+^ entry. Upon depletion of Ca^2+^ stores induced by TG, a sarco/endoplasmic reticulum Ca^2+^-ATPase pump inhibitor, we analyzed Ca^2+^ influx patterns in non-treated, 1-hour, and 24-hour quercetin-treated mast cells, as shown in Fig 4b. Under conditions without extracellular Ca^2+^, a slight increase in the RFU340/380 ratio, reflecting the depletion of intracellular Ca^2+^ stores, was observed. Upon the addition of extracellular Ca^2+^, sustained Ca^2+^ influx was detected, indicating Ca^2+^ entry through open ORAI channels. Ca^2+^ influx was quantified using the indexes of peak influx and influx rates. Both the peak Ca^2+^ influx and the influx rate within the initial 15 s were significantly suppressed in 1-hour quercetin treated RBL-2H3 cells compared to those in control cells (Fig 4c and 4d). Notably, 24-hour treatment with quercetin even elevated store-operated Ca^2+^ entry, indicated by a 9.51% increase in peak Ca^2+^ influx compared to non-treated cells. Taking these results together, enhancement of Ca^2+^ influx was observed in prolonged quercetin-treated RBL-2H3 cells, which differs from the inhibitory effect of quercetin in cells with short-term exposure.

### Varied treatment durations of quercetin differently affected IgE-mediated response of RBL-2H3 cells

**Fig 5:**
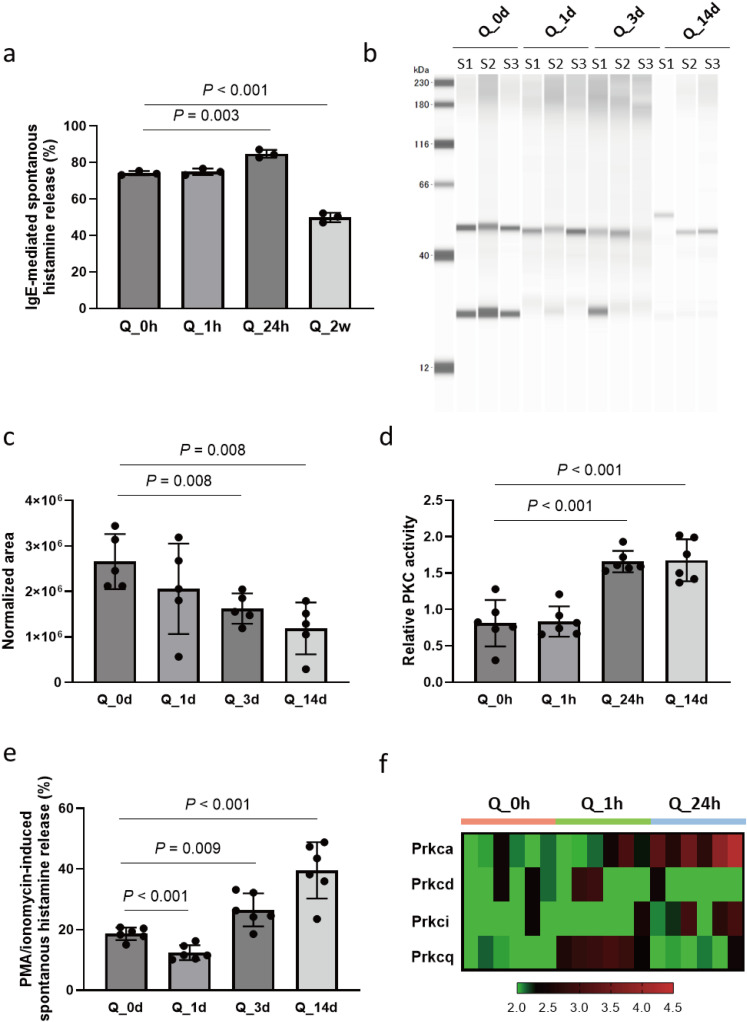
Functional evaluation of FcεRI/PKC/Ca^2+^ signaling pathway. (a) Bioassay of IgE-dependent histamine release from quercetin-treated RBL-2H3 cells for indicated durations (1 hour (Q_1h), 24 hours (Q_24h), and 14 days (Q_2w)) and non-treated cells (Q-0h). (b) Expression of FcεRI (MW: 45-65 kD) in quercetin-treated RBL-2H3 cells for indicated durations and non-treated cells. Immunoblot assay of protein extracts from cells was analyzed using a Jess automated simple western blotting system. Representative images of sample (S) 1–3 are shown. (c) Quantitative results of normalized expression of FcεRI. Results are expressed as mean ± S.D. (n = 6). (d) Intracellular PKC activity assay in quercetin-treated RBL-2H3 cells for indicated durations and non-treated cells. Results are expressed as mean ± S.D. (n = 6). (e) Bioassay of PKC-triggered histamine release from quercetin-treated RBL-2H3 cells for indicated durations followed by PMA/ionomycin stimulation. Results are expressed as mean ± S.D. (n = 6). (f) Heatmap depicting normalized expression of genes encoding PKC isozymes in quercetin-treated and non-treated mast cells (n = 6).

In addition to verifying the efficacy of quercetin on Ca^2+^ entry, we also explored its effects on the IgE-mediated signaling pathway. Initially, we evaluated histamine release triggered by DNP-BSA in mast cells treated for various durations. To assess the responsiveness of mast cells to long-term exposure to quercetin, we included an extended treatment duration of 2 weeks in the subsequent time series studies. In parallel with TG-induced hexosaminidase, IgE cross-linkage-induced histamine release from 1-hour quercetin treated mast cells was elevated compared to that in control cells (Fig 5a). On the other hand, 2-week treatment with quercetin significantly suppressed IgE-mediated cell activation. We then evaluated the protein expression level of FcεRI, considering that changes in protein expression typically follow transcriptomic alterations with a temporal delay (Figs 5b and 5c and S2).

Western blot analysis was conducted on samples with different treatment durations: 1 day (short-term treatment), 3 days (prolonged treatment), and 14 days (long-term treatment). The expression level of FcεRI was significantly down-regulated by constant quercetin treatment for 3 days and 14 days. Quercetin did not affect the expression level of FcεRI in 1 day quercetin-treated cells.

This led to the intriguing question, how do mast cells exhibit a hyperresponsive reaction to antigenic stimuli with reduced input from stimulatory signals? By examining the transcriptomic profile of the FcεRI pathway (Fig 3b), we discovered a potential functional alteration of PKC in mast cells treated with quercetin for 1 hour. Surprisingly, our PKC activity assays revealed significantly elevated cytoplasmic PKC activity in mast cells treated with quercetin for 1 hour and 14 days compared to control cells. Given that quercetin is known to be a notable protein kinase inhibitor, the mechanism behind the increased PKC activity in mast cells exposed to quercetin over extended periods remains unclear. To confirm and functionally validate this finding, we used phorbol ester (PMA) and a calcium ionophore (ionomycin) to stimulate calcium-sensitive PKC isozymes (α and β). We observed a significant increase in PMA/ionomycin-induced histamine release in mast cells treated with quercetin for 1 hour and 14 days compared to control cells. This parallel increase in PKC activity and histamine release suggests a novel and unexpected modulatory role of quercetin in PKC function in mast cells. Finally, the transcriptome profile obtained from the RNA-seq study was re-explored, and individual expression levels of PKC isoforms at the mRNA level in each sample were plotted on a heatmap (Fig 5f). Compared to non-treated cells, the expression of *Prkaca*, encoding PKCα, showed a gradually increasing tendency in a treatment duration-dependent manner.

Taken together, these findings indicate that prolonged treatment with quercetin suppressed IgE cross-linkage-induced activation and downregulated FcεRI. However, through unknown mechanisms, prolonged exposure to quercetin potentially elevated intracellular PKC activity.

## Discussion

In this study, we investigated the multifaceted effects of quercetin, a well-known dietary flavonoid with the ability to inhibit protein tyrosine and serine/threonine kinases, on RBL-2H3 cell responsiveness over varying exposure durations. Our findings revealed that quercetin initially reduced mast cell activity with short-term treatment. However, with prolonged exposure, quercetin transiently enhanced both IgE cross-linkage-mediated and non-IgE-mediated responses. Consequently, prolonged quercetin treatment downregulated IgE-mediated degranulation and FcεRI expression, while potentially sensitizing RBL-2H3 cells to other non-IgE secretagogues through enhanced PKC activity.

The ability of quercetin to inhibit mast cell degranulation is well documented, demonstrating strong inhibitory effects on the release of histamine, interleukin (IL)-8, and chemoattractant protein-1 in compound 48/80 or IgE/antigen-stimulated human mast cells [14,15]. These studies show that quercetin can inhibit IgE- or Mas-related G protein-coupled receptor X2 activation by suppressing the Lyn/PLCγ/IP3R/Ca^2+^ signaling cascade at a dose of 200μM during short-term treatment (30 min). Additionally, in human umbilical cord blood-derived cultured mast cells, 45-minute exposure to 100μM quercetin significantly suppressed the release of histamine, IL-6, TNF-α, and tryptase, along with intracellular Ca^2+^ level and phosphorylation of PKCθ [16]. The feasibility and efficacy of quercetin have been demonstrated in models of allergic and chronic inflammatory diseases, such as asthma, rhinitis, and arthritis [17,18]. These findings suggest that quercetin is a promising candidate for clinical applications.

Alternatively, a sensitization effect of quercetin, not as a suppressor, on RBL-2H3 cells responding to polybasic secretagogues has been reported [7]. In this report, after prolonged exposure to quercetin for 2 days at a dose of 30μM, RBL-2H3 cells responded to compound 48/80 stimulation, which did not occur in non-treated cells, via activation of the Gi-protein/PLCβ3/Ca^2+^/PKC cascade. The capability of quercetin to elevate PKC activity was also reported by Rossel et al. [19]. Under overexpression of PKCα, PKC activity increased 12-fold in response to the combination of treatment with quercetin and anti-CD95 for over 12 hours in a HPB acute lymphoblastic leukemia cell line. In the present study, we observed an increase in PKC activity and a decrease in FcεRI expression in RBL-2H3 cells treated with 3μM quercetin for an extended period. IgE-mediated activation of these cells showed a transient increase after 24 hours of treatment but was significantly suppressed after long-term treatment (2 weeks). Conversely, after initial inhibition with short-term treatment, the cells exhibited sustained hyperresponsiveness to PKC triggers (PMA/Iono) from 3 days to 2 weeks of treatment.

The apparent conflicting conclusions on the role of quercetin in RBL-2H3 cell reactivity might be due, at least in part, to the existence of several PKC isoenzymes. Among eleven reported PKC isozymes, conventional (α, β1, β2, γ), novel (δ, ε, η, θ), and atypical (ζ, ι, λ) isozymes depending on their requirement for the cofactor Ca^2+^, diacylglycerol, and phosphatidylserine are involved [20]. The conventional PKC-α and PKC-β isoforms primarily contribute to the immediate degranulation response upon receptor-mediated signaling, such as through the FcεRI receptor upon IgE cross-linking with antigens [21]. They are implicated in the phosphorylation of downstream signaling molecules involved in the exocytosis of granules containing histamine and other mediators, and mediate the rapid release of preformed granules containing histamine and other inflammatory mediators. Novel PKC-δ and PKC-ε play roles in the sustained and late phases of mast cell activation. They are involved in the regulation of gene expression, production of cytokines, and modulation of cell survival and apoptosis. Some atypical PKCs, such PKCι, also exhibit high immunoreactivity [20]. The complexity of PKC isozymes in cellular functional regulation is well recognized. Some PKC isozymes positively regulate cell functions, while others negatively regulate the same functions. The observed outcomes represent a balance within the isozyme network. Additionally, due to the structural and functional similarities between different PKC isozymes, one isozyme can often compensate for the loss or inhibition of another, resulting in functional redundancy [22]. Although the specific functional roles of each isozyme are still being elucidated, the significant elevation of PKC-α and PKC-ι observed in our gene expression profile may be a compensatory feedback response to the inhibition of other PKC isozymes by long-term exposure to quercetin. Furthermore, while RBL-2H3 cells are widely used as a model due to their accessibility and functional similarity to mast cells, we recognize that they may not fully replicate all aspects of primary mast cell biology, such as the diversity of protein expression and signaling pathways observed in different mast cell subtypes. This limitation underscores the need for caution when extrapolating findings to mast cells in vivo and highlights the importance of validating key findings in primary mast cell sources or in vivo models in future studies. A more detailed evaluation of the distinct effects of quercetin on the expression and function of each PKC isozyme, using pharmacological inhibitors or knockdown approaches in different tissue-derived primary mast cells, would provide deeper insights into the efficacy of quercetin in modulating mast cell activation and its broader implications for mast cell biology.

The transient hyperresponsiveness and sensitization of mast cells due to quercetin exposure could explain its clinical impacts. When quercetin is used as a mast cell stabilizer and immunological modulator in managing chronic allergic and inflammatory diseases, there is a potential for symptom relapse due to this transient hyperresponsiveness. While quercetin can suppress antigen-triggered mast cell activation with continuous exposure, high PKC activity may cause sensitized mast cells to overreact to polybasic secretagogues or PKC agonists, rather than specific antigens via FcεRI. This overreaction could lead to modulation of mast cell status in innate immune responses and secondary onset of local or systemic mast cell activation syndromes. IgE-independent stimuli, such as ligands of certain G-protein-coupled receptors (e.g., muscarinic M1 receptor, adenosine A3 receptor, Mas-related G-protein coupled X2 receptor), polybasic molecules, neuropeptides, complement fragments, and physical stimuli, could induce hyperresponsiveness of sensitized mast cells via hyperresponsiveness of PKC/Ca^2+^ cascades [5,23].

Maybe because of quercetin’s excellent oral tolerance, most human intervention studies lack detailed information on adverse events or simply state their occurrence without specifics [24]. This makes it difficult to determine the potential risk of mast cell activation syndrome in quercetin consumers. Previous reviews have noted hypersensitivity reactions such as skin rashes, itching, and other allergy-like symptoms as potential adverse effects of quercetin [25–28]. Transient symptoms, including prolonged pain at the injection site, dyspnea, and emesis, were also reported in subjects receiving intravenous quercetin at a dose of 51.3 mg/kg (Ferry et al., 1996). It remains unclear whether these side effects are related to irritated mast cells, and whether they are dose-dependent or treatment duration-dependent [29].

Quercetin has garnered significant scientific interest due to its immunoprotective, anti-inflammatory, and anticarcinogenic properties. Recently, it is even recognized as a leading senolytic agent, targeting fundamental aging processes to delay, prevent, or alleviate multiple age-related diseases and disorders simultaneously, rather than addressing them individually [30,31]. With the accumulating evidence supporting its therapeutic potential, widespread clinical use of quercetin can be predicted. Given quercetin’s multifaceted effects on intracellular signaling pathways in RBL-2H3 cells, future research should aim to optimize its benefits and establish a comprehensive safety profile, particularly concerning long-term use of high supplemental doses. Ongoing studies should focus on determining optimal outcomes and dosing regimens, documenting adverse effects, and assessing clinical safety parameters for future therapeutic interventions.

## Supporting information

S1 Fig Cell viability and proliferation were monitored in quercetin-treated RBL-2H3 cells for (a) 1 hour, (b) 24 hours, and (c) 14 days treatment duration. (TIF)

S2 Fig Raw image of western blot assay. Expression of FcεRI (MW: 45-65 kD) in 1 day(Q_1d), 3 days (Q_3d), and 14 days (Q_14d) quercetin-treated RBL-2H3 cells and non-treated cells (Q_0d). Immunoblot assay of protein extracts from cells was analyzed using a Jess automated simple western blotting system. Images of 6 samples per group are shown.
(PDF)
