# Supplementary figures and images for: Time-sensitive effects of quercetin on rat basophilic leukemia (RBL-2H3) cell responsiveness and intracellular signaling
Source: PLoS One. 2025 Feb 24;20(2):e0319103. doi: 10.1371/journal.pone.0319103 (PMC11849837; doi:10.1371/journal.pone.0319103)

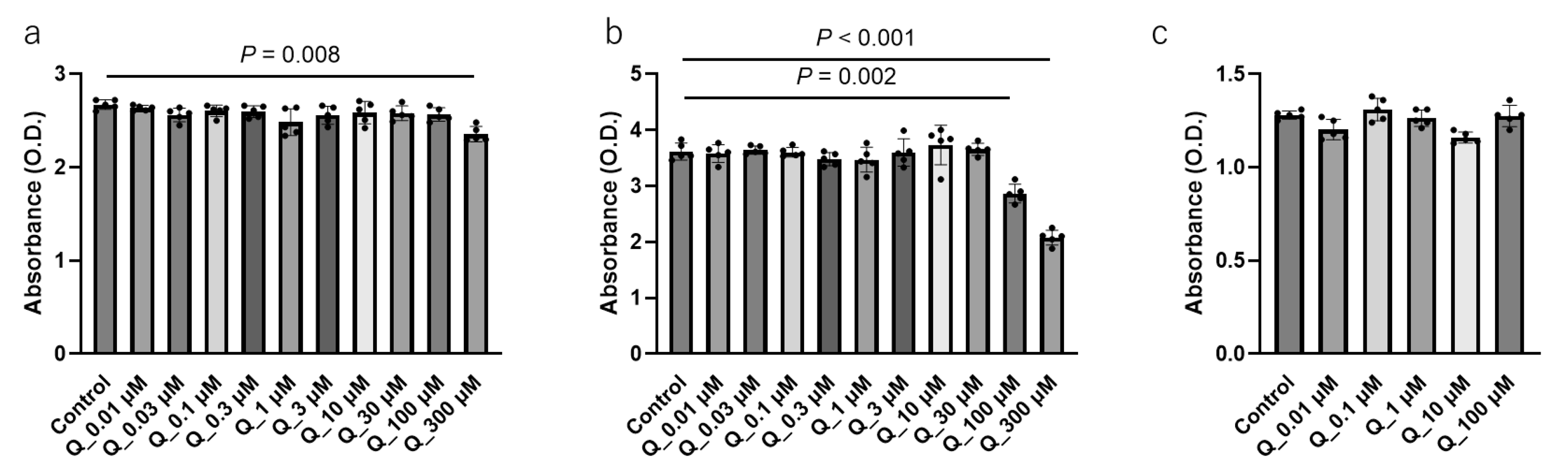

Supplement: S1 Fig — (TIF) [file pone.0319103.s001.tif]

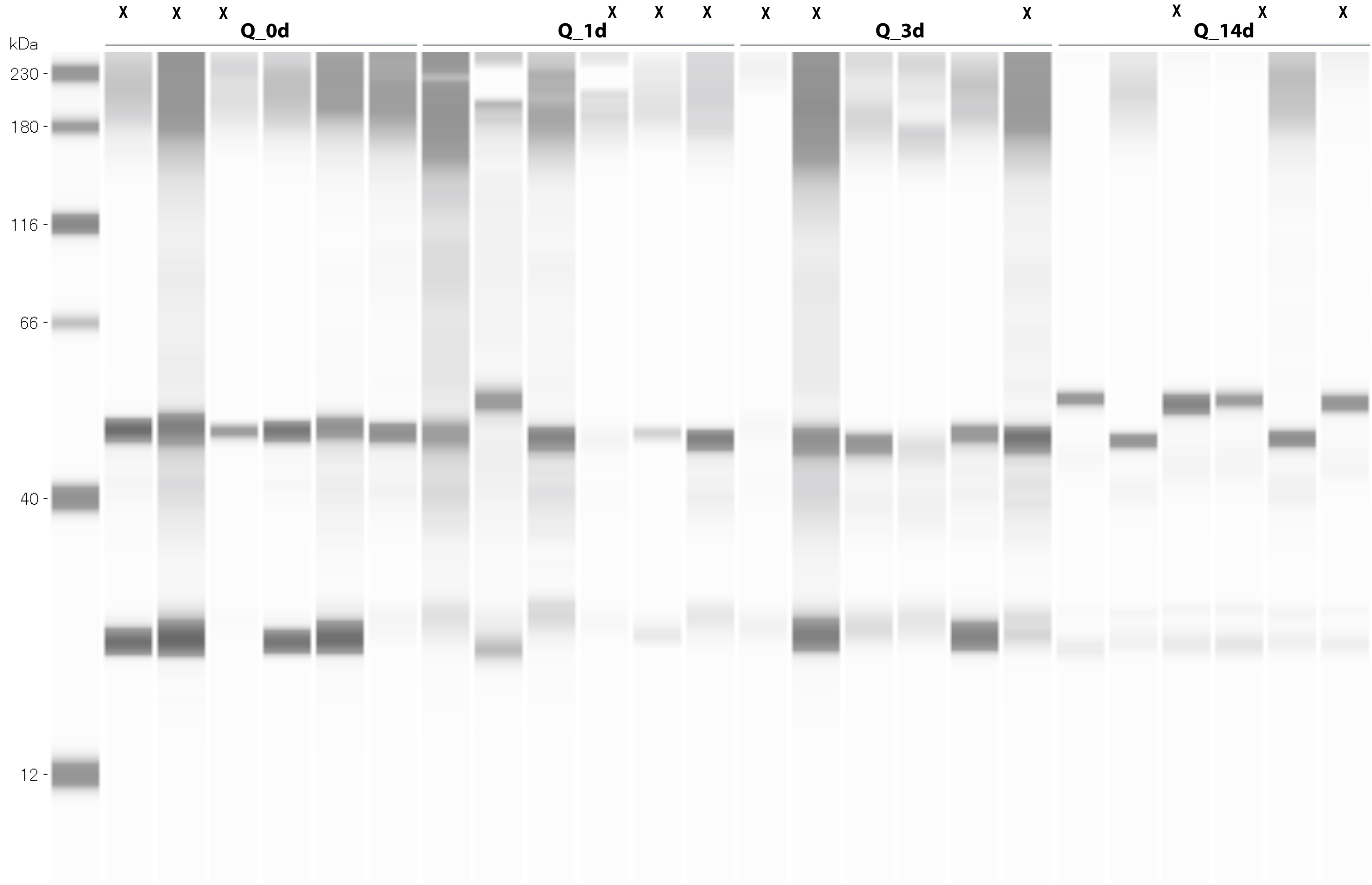

Supplement: S2 Fig — Expression of FcεRI (MW: 45-65 kD) in 1 day(Q_1d), 3 days (Q_3d), and 14 days (Q_14d) quercetin-treated RBL-2H3 cells and non-treated cells (Q_0d). Immunoblot assay of protein extracts from cells was analyzed using a Jess automated simple western blotting system. Images of 6 samples per group are shown. (PDF) [file pone.0319103.s002.pdf]
